# Supplementary material for: Deletion of wfs1 Impairs Oligodendrocyte Precursor Cells Dorsal Distribution and Myelination Through the wfs1-hmgcs1 Axis in Zebrafish
Source: Biology (Basel). 2026 Aug 16;15(16):1402. doi: 10.3390/biology15161402 (PMC13510151; doi:10.3390/biology15161402)
Supplement: Supplementary file 1 [file biology-15-01402-s001.zip › File S1-Transcriptome sequencing data and differentially expressed genes (DEGs) in wfs1-- zebrafish at 5 dpf/1.GO/wfs1KOvsWT/GSEA/WT/SEMAPHORIN_RECEPTOR_ACTIVITY/SEMAPHORIN_RECEPTOR_ACTIVITY(GO_0017154).html]

Details for gene set SEMAPHORIN\_RECEPTOR\_ACTIVITY(GO:0017154)[GSEA]


|  || Dataset | deg.sample |
| Phenotype | sample.cls |
| Upregulated in class | WT |
| GeneSet | SEMAPHORIN\_RECEPTOR\_ACTIVITY(GO:0017154) |
| Enrichment Score (ES) | -0.6181543 |
| Normalized Enrichment Score (NES) | -1.8616517 |
| Nominal p-value | 0.0 |
| FDR q-value | 0.23588744 |
| FWER p-Value | 0.538 |
Table: GSEA Results Summary

  

Fig 1: Enrichment plot: SEMAPHORIN\_RECEPTOR\_ACTIVITY(GO:0017154)
  
Profile of the Running ES Score & Positions of GeneSet Members on the Rank Ordered List

  

| PROBE | DESCRIPTION   (from dataset) | GENE SYMBOL | GENE\_TITLE | RANK IN GENE LIST | RANK METRIC SCORE | RUNNING ES | CORE ENRICHMENT || 1 | 100150664 | met |  |  | 5592 | 0.627 | -0.1152 | No |
| 2 | 100333657 | plxnb2b |  |  | 9204 | 0.353 | -0.1945 | No |
| 3 | 402973 | nrp1b |  |  | 10932 | 0.248 | -0.2249 | No |
| 4 | 561433 | plxnb2a |  |  | 11165 | 0.235 | -0.2096 | No |
| 5 | 561285 | plxna2 |  |  | 11803 | 0.201 | -0.2103 | No |
| 6 | 566546 | plxnb3 |  |  | 12800 | 0.150 | -0.2271 | No |
| 7 | 405902 | nrp2b |  |  | 13133 | 0.134 | -0.2246 | No |
| 8 | 325938 | plxna4 |  |  | 13702 | 0.107 | -0.2322 | No |
| 9 | 567422 | plxna3 |  |  | 15131 | 0.040 | -0.2731 | No |
| 10 | 795761 | plxna1a |  |  | 17037 | -0.034 | -0.3296 | No |
| 11 | 558065 | plxna1b |  |  | 18123 | -0.082 | -0.3558 | No |
| 12 | 405901 | nrp2a |  |  | 18963 | -0.120 | -0.3706 | No |
| 13 | 353246 | nrp1a |  |  | 19267 | -0.134 | -0.3672 | No |
| 14 | 561012 | plxnb1a |  |  | 19539 | -0.145 | -0.3618 | No |
| 15 | 570222 | plxnb1b |  |  | 26891 | -0.619 | -0.5329 | Yes |
| 16 | 402998 | plxnd1 |  |  | 29609 | -1.098 | -0.5126 | Yes |
| 17 | 562273 | plxnc1 |  |  | 30870 | -1.828 | -0.3762 | Yes |
| 18 | 561237 | si:dkey-183c2.4 |  |  | 30932 | -1.894 | -0.1960 | Yes |
| 19 | 100034640 | si:ch211-127b11.1 |  |  | 31262 | -2.350 | 0.0197 | Yes |
Table: GSEA details
[plain text format]

  

Fig 2: SEMAPHORIN\_RECEPTOR\_ACTIVITY(GO:0017154)
  
Blue-Pink O' Gram in the Space of the Analyzed GeneSet

  

Fig 3: SEMAPHORIN\_RECEPTOR\_ACTIVITY(GO:0017154): Random ES distribution
  
Gene set null distribution of ES for
 **SEMAPHORIN\_RECEPTOR\_ACTIVITY(GO:0017154)**
